# Investigating the sensitivity difference of gaseous and particulate carbon in two-phase sample transport in LA-ICP-MS†

**DOI:** 10.1039/d5ja00172b

**Published:** 2025-06-30

**Authors:** Lukas Brunnbauer, David Ken Gibbs, Detlef Günther, Andreas Limbeck

**Affiliations:** a TU Wien, Institute of Chemical Technologies and Analytics Getreidemarkt 9/164 1060 Vienna Austria lukas.brunnbauer@tuwien.ac.at; b ETH Zurich, Laboratory of Inorganic Chemistry, Department of Chemistry and Applied Biosciences, ETH Zurich Vladimir-Prelog-Weg 1 8093 Zürich Switzerland

## Abstract

LA-ICP-MS is a widely used analytical technique for elemental analysis of different solid samples, including carbon-based samples. To compensate for matrix-effects and instrumental drifts during analysis, application of an internal standard is recommended. For carbon-based samples, the application of carbon as an internal standard seems reasonable but is typically not recommended due to the so-called two-phase sample transport where ablated carbon is transported both as particulate and gaseous species. The quantitative deviations in the sensitivity of particulate and gaseous carbon have not been accessible so far but would provide useful insights into the application of carbon as an internal standard. More precisely, if similar sensitivity for particulate and gaseous carbon species is found, application as an internal standard would not be restricted. To investigate this, we analyze the two-phase sample transport of carbon upon ablation of 5 different polymers, which all form different ratios of particulate and gaseous carbon species. Amongst the studied materials, it has been observed that 2 samples provide almost exclusive formation of a gas phase. Correlating these observed signals for selected polymers with the ablated mass of carbon allows us to calculate the sensitivity of gaseous carbon species as 13.8 cts per pg. Using a mass balance approach, we estimated the sensitivity of particulate carbon for the 3 other polymers, where we find significant differences in sensitivity ranging from 1.69 cts per pg to 14.06 cts per pg. This indicates that the sensitivity for particulate carbon species is highly dependent on the sample matrix, resulting in sensitivity differences up to a factor of 7. All in all, the findings of this study support the results of carbon being an inadequate choice for an internal standard in LA-ICP-MS.

## Abstract 2

Quantitative assessment of the sensitivity difference of gaseous and particulate carbon in LA-ICP-MS.

## Graphical abstract

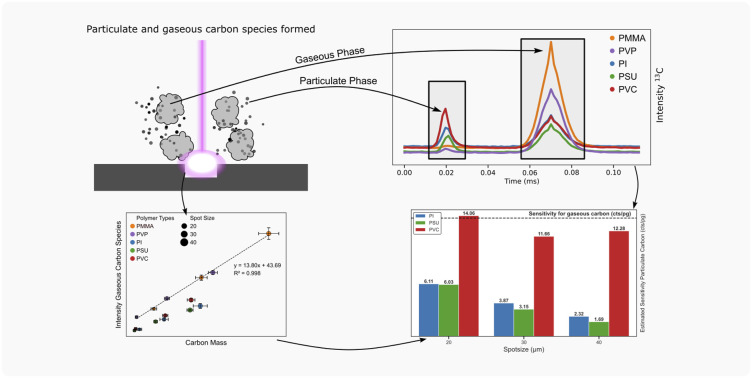

## Introduction

LA-ICP-MS is an analytical technique used for elemental analysis applied in a wide range of different fields. Starting out in the field of geology^1^ where it is still widely applied today,^2^ biological samples^3^ and materials science^4,5^ have become important application fields, demonstrating the technique's versatility. Besides providing several advantages such as outstanding sensitivity and access to isotopic ratios, some challenges such as susceptibility to matrix effects must be considered. In the last few decades, different approaches have been developed to compensate for matrix effects providing more accurate results. Besides standard bracketing, the application of an internal standard (typically a matrix-element with a known concentration) is becoming a pre-requisite to compensate for instrumental drifts and variations in ablation, transport, vaporization, atomization, and ionization in the ICP.^6,7^ In the case of carbon-based samples such as minerals and rocks, hard and soft tissues, as well as cells and natural or synthetic polymers, the use of carbon as an internal standard would be the obvious choice.

However, in previous studies, it was found that carbon experiences a phenomenon called two-phase sample transport, where part of the carbon mass is transported as gaseous species and another part is transported as particulate matter, which hinders its application as an internal standard. This phenomenon was first observed by Todoli *et al.*^8^ demonstrating that after ablation of PVC, both gaseous and particulate carbon species are formed. This effect was investigated in more detail by Frick *et al.*,^9^ who used a filter and a gas exchange device to separate the particulate and gaseous species. It was found that the ratio of gaseous and particulate carbon formed after LA is matrix-dependent with some relation to the oxygen content of the sample. Additionally, they found that other elements under investigation were only present in the particulate phase. Therefore, the authors concluded that normalization to carbon as an internal standard is not applicable and will not fully compensate for variations in the laser ablation process, transport of the ablated material towards the ICP, as well as vaporization, atomization and ionization in the ICP. Furthermore, Frick *et al.*^9^ provided an overview of literature where either carbon was used as an internal standard or specifically not used which highlights the controversy of this topic.

Recent improvements in instrumentations such as the introduction of rapid response cells and next generation ICP-TOF-MS and ICP-Q-MS^10^ enable the measurement of so-called single pulse response (SPR) profiles representing the transient signal of a single laser shot.^11–13^ This allows for the investigation of the effect of two-phase sample transport directly in more detail without the need to employ filters or gas exchange devices. In a study by van Helden *et al.*,^14^ the authors found that upon ablation of gelatine, both carbon and some other elements exhibited two-phase sample transport. Additionally, they showed that the ratio of gaseous carbon grows with increasing laser energy used for ablation. In a follow-up study, the authors investigated the effect of this two-phase transport on image quality in LA-ICP-MS imaging,^15^ revealing substantial degradation of image quality for elements experiencing two-phase sample transport.

In general, it is expected that gaseous and particulate carbon species show variations in signal response due to differences in transport efficiency as well as differences in vaporization, atomization and ionization efficiency. Compared to particulate species, better atomization and ionization in the ICP are expected for gaseous species since vaporization is not required. Moreover, gaseous species are expected to be transferred from the ablation chamber to the ICP with close to 100% transport efficiency, whereas transport efficiencies reported for particle aerosols in LA range from 8% to 77% (ref. 16–18), depending on the instrumentation used (*e.g.*, pulse width) and ablation atmosphere. Thus, the use of carbon for signal normalization introduces an additional source of error, especially if the sample of interest consists of different carbon containing compounds, since the partitioning between gas and particle phase might vary within the sample matrix. Some examples of carbon-containing matrices, which would suffer from this effect and are commonly analyzed in LA-ICP-MS, include biological and medical samples such as tissues, cells, and plants, or samples from the field of materials science such as polymers or composites.

In this work, we aim to quantify the two-phase sample transport of carbon and compare the sensitivity of gaseous carbon species with the sensitivity of particulate carbon. Therefore, we analyze 5 different polymer types (polyimide (PI), poly(methyl methacrylate) (PMMA), polysulfone (PSU), polyvinyl chloride (PVC), and polyvinylpyrrolidone (PVP)) with a constant laser energy of 5.8 J cm^−2^ and three different laser spot sizes (20, 30, and 40 μm).

## Experimental

### Sample preparation

High-purity silicon wafers (n-doped) used as substrate materials were provided by Infineon Austria AG (Villach, Austria). Polyimide-based P84 in powder form (>98% purity) was obtained from HP Polymer GmbH (Lenzing, Austria). PMMA, PSU, PVC, and PVP in powder form were obtained from Acros Organics (Geel, Belgium). Information about the carbon content and density of each polymer was provided by the manufacturer. For the preparation of polymer samples, each polymer was dissolved in *N*-methyl-2-pyrrolidone (NMP, >99.7%, Sigma-Aldrich), yielding 10 wt% solutions. Polymer films were prepared by drop-casting 40 μL of the corresponding solution on a 10 mm × 10 mm silicon wafer. Samples were cured on a hotplate at 80 °C for 60 min to remove the NMP. The procedure described results in polymer films with a thickness in the range of 10 μm with slight variations depending on the polymer type. The center region of the films (approx. 7 mm × 7 mm) was mostly flat, allowing for reproducible sampling.

### Measurement setup

LA-ICP-MS analysis was carried out using an imageGEO193 LA system (ESL, Bozeman, Montana, US) equipped with an ArF excimer laser (ExciStar 200, Coherent Laser Systems, Göttingen, Germany) emitting at 193 nm with a pulse width of 7 ns. The system was equipped with an imaging cup in combination with a TV3 ultrafast washout cell and He was used as a carrier gas. The flows (chamber and cup He) were optimized for highest signal intensities and clear separation of the particulate and gaseous carbon to facilitate data evaluation. The gas flows affect the delay between the particulate and gaseous carbon peaks with increasing gas flows resulting in a shorter delay. It should be noted that no experimental conditions were found where the particulate and gaseous carbon peaks arrive at the ICP-MS at the same time. The LA system was connected to a NexION 5000 ICP-MS (PerkinElmer, Waltham, Massachusetts, US) using a PEEK capillary with an inner diameter of 0.03′′ and a length of 100 cm with a dual concentric injector (DCI) (ESL, Bozeman, Montana, US). Ar was added to the He stream right before the ICP in the DCI to avoid peak broadening. The ICP-MS was tuned daily for maximum ^115^In^+^ intensity while keeping ^232^Th^16^O^+^/^232^Th^+^ below 1% when ablating the NIST612 glass standard (Standard Reference Material, National Institute of Standards and Technology, Gaithersburg, MD). ICP-MS data were collected using Syngistix 3.5 using the NanoApplication (version 3.5) enabling single *m*/*z* monitoring without a settling time of the quadrupole which is beneficial when recording short transient signals with high time resolution. A summary of LA-ICP-MS measurement conditions is provided in Table 1. Obtained signal intensities are given in counts (cts) in all following discussions.

**Table 1:** LA-ICP-MS measurement parameters

imageGEO193		NexION5000	
Laser fluence (J cm^−2^)	5.8	RF power (W)	1600
Chamber/cup He (ml min^−1^)	250/200	Ar make-up gas (L min^−1^)	1.2
Spot size (circular) (μm)	20, 30, 40	Detected isotope	^13^C^+^
Repetition rate (Hz)	10	Dwell time (ms)	0.3

Analysis was performed using 101 shots fired with a distance of 20 μm between each laser shot. Using these conditions, each single laser shot resulted in one ablation crater and the corresponding single-pulse-response signal was not influenced by preceding shots.

Crater volumes were determined using a Dektak XT stylus profilometer (Bruker Corporation, MA, USA), measuring 3D maps with a resolution of 5 μm in the *x*-direction and 0.07 μm in the *y*-direction. Obtained craters showed regular spherical shapes with uniform depth for all polymers. The volume of the ablation craters was assessed using the open-source software Gwyddion 2.67.^19^ A detailed description of the crater shapes and the data evaluation process is provided in the ESI.† The ablated mass for each LA parameter was calculated from the crater volume as well as the known density and carbon content of the individual polymers.

### Data evaluation

A python-based Jupyter notebook was developed for data processing and evaluation in order to perform the following steps: (1) background correction by subtracting the average background signal for ^13^C^+^ for each measurement; (2) application of scipy.signal.find_peaks^20^ to identify peak positions of the particulate and gaseous peaks in the transient signal; (3) calculation of the sum intensity of the particulate and gaseous peaks for further data evaluation. In the case of PMMA and PVP, where the formation of mainly gaseous carbon was observed, it was not possible to directly identify the position of the potential particulate peak for individual SPRs. Therefore, for these two samples, in a first step, transient signals were averaged enabling the estimation of the time difference between the gaseous and particulate peaks. This allowed us to estimate the position of the particulate peak relative to the gaseous peak for the individual SPR signals.

## Results

In this work, we investigate and quantify the difference in sensitivity of gaseous carbon species and particulate carbon upon ablation of various polymers, showing distinct differences in the formation of gaseous phase and particulate material. Characterization of the resulting craters using a profilometer allows us to precisely determine the carbon mass that was ablated and is expected to generate the signal in the ICP-MS. With this approach and in case one polymer forms a gas phase only, a calibration for carbon can be constructed which could be used to assess the variation in sensitivity of gaseous carbon species and particulate carbon.

### Qualitative single-pulse-response (SPR)

**Fig. 1:**
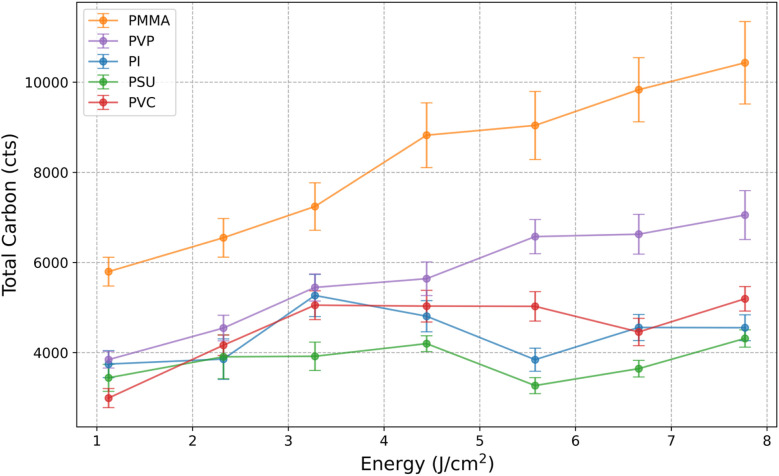
Total carbon signal for the investigated polymers for different laser energies with a spot size of 30 μm (*n* = 101 shots).

In an initial experiment, polymer samples were measured with a constant spot size of 30 μm and varying laser energy (1.1–7.8 J cm^−2^). For all investigated laser energies and polymers, two peaks in the transient signal were detected stemming from the two-phase sample transport of carbon. A general trend of increase in the ratio of the particulate species with decreasing laser energies is observed which is also described by van Helden *et al.*^14^ PMMA and PVP show almost exclusive formation of gaseous species at 5.8 J cm^−2^. By evaluating the total carbon signal (Fig. 1), we find an increase for PMMA and PVP with increasing laser energy whereas PI, PSU, and PVC show little variations.

**Fig. 2:**
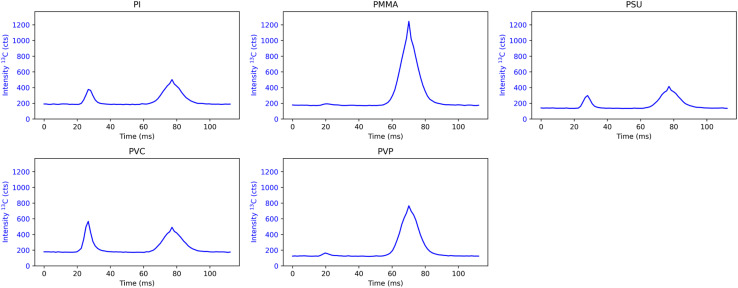
Averaged SPRs for the 5 polymer types upon ablation with a 30 μm spot size and 5.8 J cm^−2^ laser energy. The first peak corresponds to particulate carbon species, whereas the second peak corresponds to gaseous carbon species. The start of the time axis is arbitrarily set to 0.

Even though 5.8 J cm^−2^ is a rather high laser energy for the analysis of polymers (indicated by the non-linear increase of the total carbon signal for some polymers), these conditions allow us to form gaseous species only for two polymers which is a precondition for the assessment of the sensitivity of gaseous carbon species. Thus, further experiments were carried out with a constant laser energy of 5.8 J cm^−2^ and spot sizes varying from 20 to 40 μm. The transient signals of the SPR (101 shots) obtained for a spot size of 30 μm are averaged and shown in Fig. 2 for qualitative assessments. For each polymer, a single laser pulse generated two separated peaks in the transient ^13^C^+^ signal, indicating two-phase sample transport of carbon. Considering that this was also observed by van Helden *et al.* upon ablation of gelatine,^14^ we can confirm that particulate carbon arrives first at the ICP-MS followed by gaseous carbon. The delay between particulate and gaseous carbon of approx. 45 ms is similar for all polymers analyzed. Additionally, peak widths for the particulate (∼15 ms) and gaseous carbon (∼25 ms) are different for all polymers investigated.

While PMMA and PVP show almost exclusive signals for gaseous carbon species, the SPRs of PI, PSU, and PVC display a significant amount of both particulate and gaseous carbon species. Additionally, total carbon signals for PMMA and PVP are significantly higher than total carbon signals for PI, PSU, and PVC. Comparing the peak integrals of gaseous and particulate carbon species for PMMA and PVP, it is found that >93% and >95% of the total carbon signal originate from the gaseous peak, respectively. In the case of the other polymers investigated, the contribution of the gaseous carbon signal accounts for 68% (PI), 70% (PSU), and 56% (PVC) of the total carbon signal.

In contrast to the work by Frick *et al.*,^9^ there does not seem to be any correlation between the oxygen content within polymers (Table 2) and the formation of gaseous carbon species. While PMMA has the highest oxygen content in its repeating unit and the majority of the total carbon signal stems from gaseous species, PVP exhibits a similar SPR despite its repeating unit only containing approximately half the amount of oxygen. In fact, the oxygen content of both PI and PSU is similar to that of PVP, but they display vastly different SPR profiles. Additionally, PVC, which contains no oxygen at all, shows a significant formation of gaseous carbon species. Nevertheless, residual oxygen in the high purity He used as a carrier gas, or oxygen outgassing from components in the ablation chamber and tubing may lead to the formation of CO/CO_2_, subsequently detected as gaseous species.

**Table 2:** Oxygen content (wt%) and oxygen atoms per repeating unit of the investigated polymers

	Oxygen content (wt%)	Oxygen atoms per repeating unit (—)
PI	18.9	Structure not known
PMMA	32.0	2
PSU	14.46	4
PVC	0	0
PVP	14.4	1

### Quantitative estimations of the sensitivity of gaseous and particulate carbon species

**Fig. 3:**
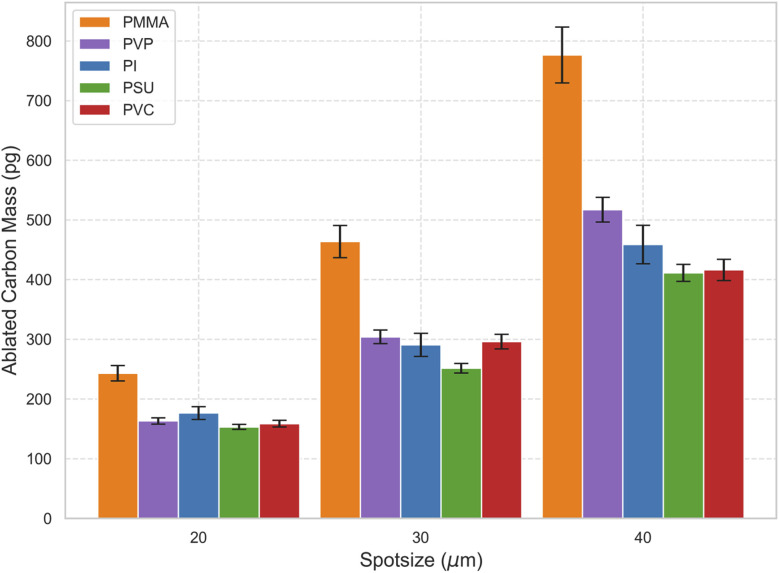
Ablated carbon masses for the different spot sizes and polymer types investigated.

Due to the exclusive formation of gaseous species for PMMA and PVP, a quantitative description of the ratios between particulate carbon and gas phase becomes possible. Therefore, knowledge about the ablated mass of carbon and hence the expected mass of carbon introduced into the ICP-MS is necessary to estimate the two mass fractions. These data were obtained by characterizing the crater volume by 3D mapping with a profilometer and calculating the ablated mass of carbon based on the crater volume, density, and carbon content of each polymer type. For each set of spot sizes and polymer type, 3 craters were analyzed resulting in a total number of 45 measured craters. Ablated masses for all spot sizes and polymer types are shown in Fig. 3. In general, an increase in the ablated mass of carbon with increasing spot sizes is observed for all polymers. PMMA showed the highest ablation rate with masses of carbon ranging from 243 pg to 777 pg for the spot sizes 20 μm and 40 μm, respectively. Shot-to-shot variations in the ablated mass of carbon based on the crater profiles range from 3.8% (PSU) to 7.7% (PI). It should be noted that we do observe deviations from the expected increase in ablated mass with increasing spot size (*i.e.*, doubling the spot size should result in an increase of ablated mass by a factor of 4). The deviations are different for the different polymers and can be attributed to the high laser energy, which as discussed previously is required to form gaseous species only for PMMA and PVP. In that case, various effects such as laser plasma shielding may limit the ablation resulting in non-linear effects.

**Fig. 4:**
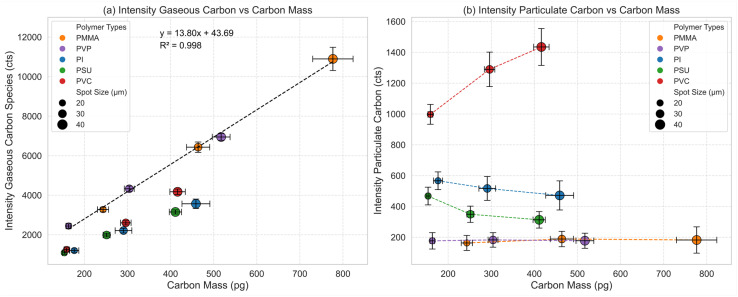
Intensity of the gaseous carbon species *vs.* carbon mass (a) and intensity of particulate carbon *vs.* carbon mass (b). Calibration for gaseous carbon obtained by calculating a linear regression for the data of PMMA and PVP. Different amounts of carbon were introduced into the ICP-MS by ablating each polymer type with different spot sizes (20, 30, and 40 μm).

Further insights into the two-phase transport of carbon can be obtained by analyzing the correlations between ablated mass of carbon and the integrated signal of both gaseous carbon (Fig. 4(a)) as well as particulate carbon (Fig. 4(b)) for all polymers. A linear relationship is found between the signal of gaseous carbon species and ablated mass of carbon for PMMA and PVP which exclusively (>93% and >95%, respectively) form gaseous carbon species (Fig. 4(a)). Since PMMA and PVP exhibit the same signal trend, we can assume that transport and atomization/ionization of the gaseous species are similar. This is confirmed by calculating an individual linear regression for the PMMA and PVP data resulting in slopes of 14.23 ± 0.01 cts per pg and 12.69 ± 0.30 cts per pg, respectively. Applying a two-sided *t*-test with one degree of freedom results in a *p*-value of 0.12, indicating that there is no reason to believe that the slopes of PMMA and PVP differ at a 5% level of significance. This outcome indicates that the chemical nature and properties of the formed gaseous species have no effect on the signal response, since observed carbon signals depend only on the introduced mass of carbon. Calculating a linear regression based on the combined data of PMMA and PVP allows us to estimate the sensitivity for the gaseous carbon based on the slope. For gaseous carbon, we determined a sensitivity of 13.8 cts per pg carbon. It should be mentioned that an intercept (43.69 cts) was observed for the obtained calibration function. This indicates either insufficient background correction or some systematic error in the determined ablated carbon masses. Nevertheless, the intercept is relatively small compared to the signals observed (varying between 2440 cts and 10 895 cts), making its influence negligible for the data evaluation.

Polymers which form both gaseous and particulate carbon species (PI, PSU, and PVC) show a lower signal for the gaseous carbon species in relation to the ablated mass of carbon than the calibration obtained for PMMA and PVP (Fig. 4(a)), since the proportion of particulate carbon is significantly higher. Nevertheless, a relationship between the detected gaseous carbon signal and the ablated carbon mass with an offset and smaller slope compared to the calibration for PMMA and PVP is found, indicating a linear increase in the formed gaseous species with increasing ablated carbon mass.

Fig. 4(b) shows the relationship between the signal for the particulate carbon species and the total ablated carbon mass. A constant low signal for the particulate carbon species in relation to the ablated carbon mass is found for PMMA and PVP. PVC shows an increase in the signal of the particulate carbon species with larger ablated mass which is the expected result. For PI and PSU on the other hand, the signal of particulate carbon species depletes slightly with increasing ablated carbon mass. This can either be explained by a decrease of the transport efficiency with increasing mass of ablated carbon or by incomplete vaporization/atomization/ionization in the ICP.

**Fig. 5:**
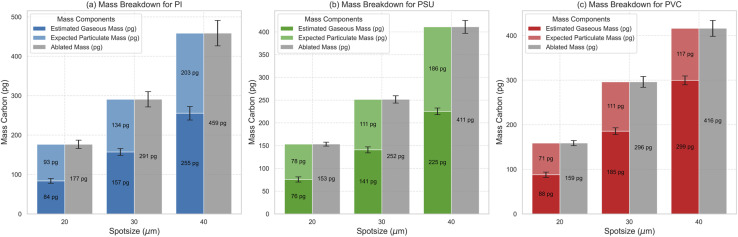
Mass balance calculations for PI (a), PSU (b), and PVC (c). Quantification of gaseous carbon is based on the obtained calibration for gaseous carbon (Fig. 4). Expected mass of particulate carbon is obtained by subtracting the quantified gaseous carbon mass from the total ablated mass (grey) determined from the crater volume.

In the next step, we can use the obtained calibration for gaseous carbon species (based on PMMA and PVP) to quantify the mass of gaseous carbon species for the three polymer types (PI, PSU, and PVC), which showed the formation of both gaseous and particulate carbon species. Since no differences in the response of the gaseous carbon species of PMMA and PVP were observed, we assume a similar transport efficiency and atomization/ionization efficiency of the gaseous species formed upon ablating the other polymers. The results are denoted by the estimated gaseous mass (pg) in Fig. 5.

Knowing the total mass of ablated carbon and due to conservation of mass (mass balance), we can now estimate the expected mass of particulate carbon by subtracting the determined mass of gaseous carbon species from the total mass of ablated carbon (Fig. 5). It should be noted that the total amount of carbon generated upon ablating PI, PSU and PVC with 20, 30 and 40 μm spot sizes is comparable, ranging from 153 pg for 20 μm to 459 pg for 40 μm, indicating an almost similar ablation behavior of these three chemically different polymers. Additionally, the expected mass of particulate carbon is in a similar range, which is surprising considering that the signal intensities for the particle phase of PVC and PSU/PI are quite different ranging from 180 cts to 1450 cts (Fig. 4(b)).

**Fig. 6:**
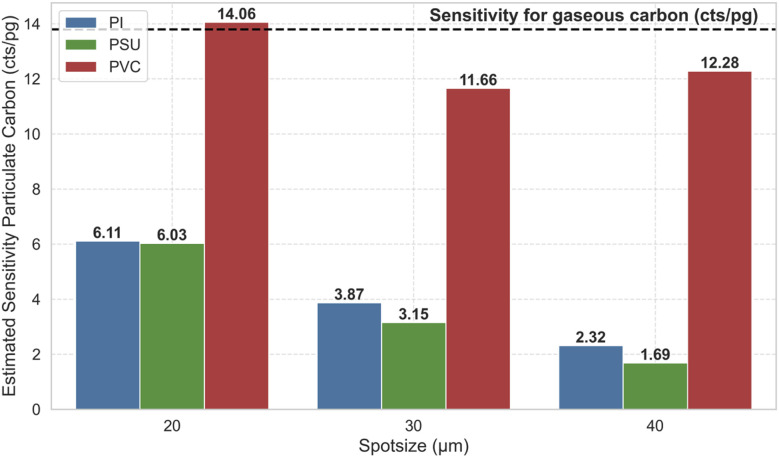
Estimated sensitivity of particulate carbon for PI, PSU and PVC for the three spot sizes 20, 30, and 40 μm in cts per pg. Additionally, the sensitivity for gaseous carbon is shown as a dashed line at 13.8 cts per pg.

We can now estimate the sensitivity for particulate carbon based on the findings in Fig. 5 by simply dividing the obtained signal for particulate carbon for PI, PSU and PVC for the different spot sizes by the expected mass of particulate carbon. The results are shown in Fig. 6.

Sensitivities for particulate carbon ranging from 1.69 cts per pg (PSU, 40 μm spot size) up to 14.06 cts per pg (PVC, 20 μm spot size) are found. While the sensitivity for particulate carbon for PVC is similar to the sensitivity for gaseous carbon of PVP and PMMA, this is not true for PI and PSU, which further display a dependence on the spot size suggesting a decrease in sensitivity with larger spot size. For a spot size of 20 μm, particulate carbon species formed upon ablation of PVC are detected with a 2.3 times higher sensitivity than PSU and PI. This discrepancy further increases for larger spot sizes up to a factor of 5.3 for PI and 7.3 for PSU (40 μm). These results indicate that the sensitivity of particulate carbon is highly dependent on the chemical nature of the analyte and is also influenced by the spot size.

Both transport efficiency and vaporization/atomization/ionization in the ICP depend on the chemical composition of the analyzed sample influencing the generated size distribution of the aerosol, formation of agglomerates and potential static charging effects. For PVC, we find sensitivities for particulate carbon species in a similar range to that for gaseous carbon species indicating that transport efficiency and vaporization/atomization/ionization are similar.

PI and PSU show a significantly lower sensitivity for particulate carbon which decreases with increasing spot size. This implies that either the transport efficiency is reduced, significant redeposition takes place, or incomplete vaporization/atomization/ionization occurs. Differences in ionization efficiency are expected to be negligible, since van Acker *et al.*^21^ reported a linear relationship between the obtained carbon signal and the particle size when analyzing polystyrene (PS) spheres up to a diameter of 20 μm. The mass of carbon introduced into the ICP-MS for a 20 μm spherical PS particle is equal to 4058 pg. Comparing this to the masses of carbon introduced in this work (<777 pg), we can assume complete vaporization/atomization/ionization for our samples, especially considering that we introduce a particle aerosol or gaseous species compared to individual particles as reported by van Acker *et al.* Therefore, we conclude that the lower sensitivity found for particulate carbon for PI and PSU is mainly caused by reduced transport efficiency from the ablation chamber to the ICP-MS or redeposition.

## Conclusion

In this work, we investigated the difference in the sensitivity of particulate and gaseous carbon species in LA-ICP-MS by analyzing 5 different polymers. While the effect of two-phase sample transport of carbon was mentioned for the first time in 1998, the difference in sensitivity was not investigated until now. Insights into the potential sensitivity differences could provide a deeper understanding of whether carbon is a suitable internal standard in LA-ICP-MS. Upon the ablation of 2 of the 5 polymer samples investigated (PMMA and PVP), we found almost exclusive formation of gaseous carbon species. We observed a linear relationship between the signal of gaseous carbon species and the ablated carbon mass for these two polymers. This allowed us to estimate the sensitivity of gaseous carbon species of 13.8 cts per pg. Using a mass balance approach enabled us to estimate the sensitivity of particulate carbon species for the 3 other polymers (PI, PSU, and PVC). Here we found significant differences in the sensitivity. While PVC showed sensitivities for particulate carbon species in a similar range to that for gaseous carbon species, PI and PSU showed a significantly lower sensitivity. Considering other reports in the literature, we conclude that the reduction in sensitivity is mainly caused by a decrease in transport efficiency. With the obtained results, we confirm that for certain matrices, there is a significant difference between the sensitivity for gaseous and particulate carbon species, hindering its application as an internal standard. Finally, it should be noted that different instrumental setups may lead to different findings due to variations in transport efficiency for particulate carbon species for different ablation cell designs. Additionally, since the formation of gaseous species may be governed by the presence of oxygen, contaminations in the used carrier gas, variations in outgassing oxygen from components in the ablation chamber and the used tubing may further alter the results.

## Conflicts of interest

There are no conflicts to declare.

## Supplementary Material

JA-040-D5JA00172B-s001

## Data Availability

The data supporting the findings of this study are available within the article or its ESI.† Other relevant data can be shared upon reasonable request.
