# Supplementary material for: Investigating the sensitivity difference of gaseous and particulate carbon in two-phase sample transport in LA-ICP-MS
Source: J Anal At Spectrom. 2025 Jun 30;40(8):2179–86. doi: 10.1039/d5ja00172b (PMC12243101; doi:10.1039/d5ja00172b)
Supplement: JA-040-D5JA00172B-s001 [file JA-040-D5JA00172B-s001.pdf]

Supplementary Information (SI) for Journal of Analytical Atomic Spectrometry.
This journal is © The Royal Society of Chemistry 2025

Supplementary information

Investigating the sensitivity difference of gaseous and particulate carbon in two-phase sample transport in LA-ICP-MS

Lukas Brunnbauer[1], David Ken Gibbs[1], Detlef Günther[2], Andreas Limbeck[1]

[1] TU Wien, Institute of Chemical Technologies and Analytics, Getreidemarkt 9/164, 1060 Vienna, Austria

[2] ETH Zurich, Laboratory of Inorganic Chemistry, Department of Chemistry and Applied Biosciences, ETH Zurich, Vladimir-Prelog-Weg 1, 8093 Zürich, Switzerland

Evaluation of the profilometer data

Profilometer data was used to assess the ablated volume for each laser shot. 3D maps were recorded covering shots of the 3 investigated spot sizes (20, 30, 40 µm) with a resolution of 5 µm in x-direction and 0.07 µm in y-direction. An exemplary map is shown in Figure S1 a) for PSU. Data was evaluated using Gwyddion 2.67. In a first step, the data is background corrected to compensate for an uneven surface or a tilt within the instrument. Therefore, the data is leveled using a polynomial function of 3rd order using Gwyddions "Polynomial Background" function (Figure S1 b)). In a next step, the laser craters are masked using Gwyddions "Mask by Threshold" function. A threshold is set accordingly to mask all the pixels corresponding to each laser crater (Figure S1 c)). Finally, the volume data is extracted by selecting each crater individually using Gwyddions "Statistical Quantities" with "including only masked region". This ensures that only pixels belonging to each crater are contributing to the obtained volume.

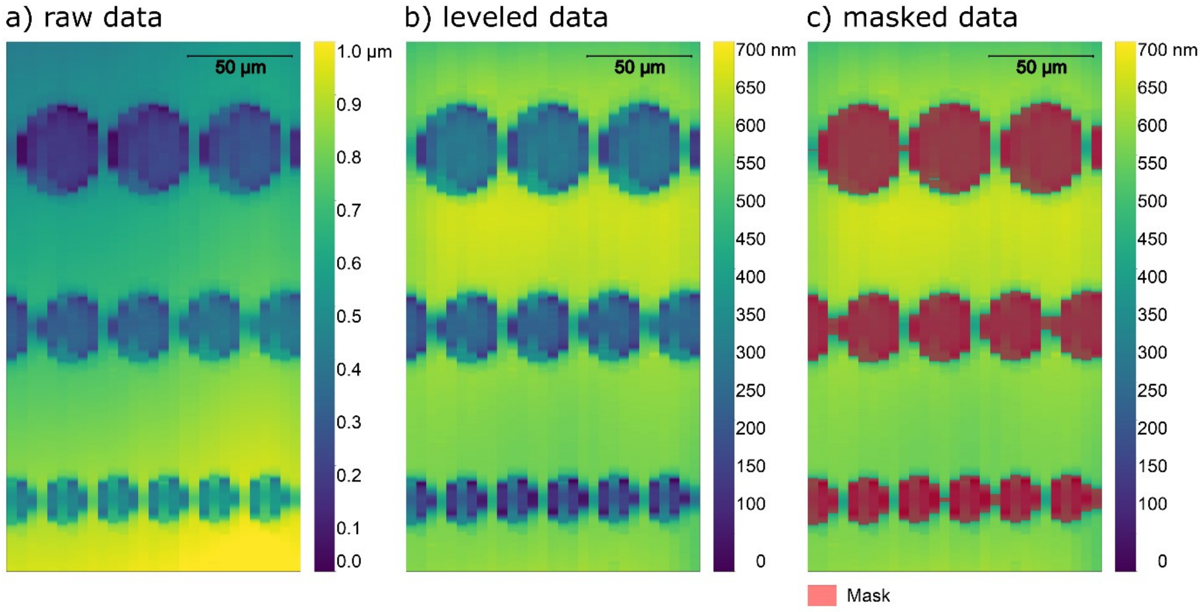

Figure S1: Step-by-step description of evaluating the profilometer data to obtain ablated volumes for the different spot sizes. Exemplary data for PSU is shown.
